# Single Incision Sling Surgery for Female Stress Urinary Incontinence: A Retrospective Cohort Single-Institution Study

**DOI:** 10.3390/jcm13164908

**Published:** 2024-08-20

**Authors:** Ayman Qatawneh, Fidaa Thekrallah, Huda M. Alaqqad, Maysa A. AlTayyar, Reem F. Ahmed, Tala O. Ashour

**Affiliations:** 1 Department of Gynecology and Obstetrics, School of Medicine, The University of Jordan, Amman 11942, Jordan; f.thekrallah@ju.edu.jo; 2 School of Medicine, The University of Jordan, Amman 11942, Jordan; hudaaqqad0301@outlook.com (H.M.A.); meso.tayyar@gmail.com (M.A.A.); reemalnuaamy95@gmail.com (R.F.A.); tashour17@yahoo.com (T.O.A.)

**Keywords:** stress urinary incontinence, mixed urinary incontinence, recurrent stress urinary incontinence, Altis^®^

## Abstract

**Background:** Stress urinary incontinence (SUI) affects around 35% of adult women and has a significant impact on quality of life. A single incision sling (SIS), such as Altis^®^, was introduced to improve original slings and avoid complications. The present study aimed to evaluate the SIS Altis^®^ subjective and objective cure rates of women with SUI, mixed urinary incontinence (MUI), recurrent SUI, and SUI with concomitant prolapse and report its complications from one single medical center. **Methodology:** A retrospective cohort, unsponsored study was conducted at the Jordan University Hospital. All women patients were treated with the SIS Altis^®^ procedure, and prolapse procedures were also completed as necessary. The chi square analysis for the cure rates was conducted between the subgroups. **Results:** From June 2016 to June 2019, 111 women patients with a mean age of 48.0 ± 11.3 years underwent a SIS Altis^®^ procedure. The overall outcome resulted in 81% and 85% of patients being subjectively and objectively cured. The subjectively cured MUI patients were significantly fewer than SUI patients (70% versus 86%, *p* < 0.05), and patients with recurrent SUI had significantly lower rates (56% for subjective and objective cure rates; *p* < 0.01 and 0.001). Of the 44 patients who underwent SIS Altis^®^ and concomitant vaginal repair surgery, no significant differences in subjective and objective cure rates were observed. Only 2.7% of women had mild pain, 2.7% had vaginal tape erosion, and 9% had to be re-operated on. **Conclusions:** The Altis^®^ procedure is effective in women who suffer from SUI for a 19-month follow-up period. However, recurrent SUI patients had lower subjective and objective cure rates than primary SUI patients. Further research is needed with a larger sample size in a prospective study design to determine the effectiveness of single SIS in patients with recurrent SUI.

## 1. Introduction

Stress urinary incontinence (SUI) is the complaint of involuntary urine leakage during effort, exertion, sneezing, or coughing [1]. The two main mechanisms behind SUI are urethral hypermobility and intrinsic sphincter deficiency [2,3]. The former mechanism occurs due to the loss of support from the pelvic floor muscles or vaginal connective tissue, and the latter mechanism occurs due to the loss of the mucosal and muscular tone in the urethra and tends to be more severe [2,3]. Furthermore, women may also have mixed urinary incontinence (MUI), which is the complaint of involuntary urine leakage associated with urgency and exertion, effort, sneezing, or coughing [1]. SUI affects around 35% of adult women and has a significant impact on quality of life [4]. Therefore, it is paramount that viable treatment options with high curative rates and low complication rates are offered to women to improve their quality of life.

When conservative measures, such as pelvic floor muscle training or electric stimulation, fail, surgery is anticipated. Several types of surgeries might be considered. However, the retro-pubic and trans-obturator sub-urethral slings are the most commonly used, despite some complications associated with passage of trocars and the mesh through the retro-pubic area and the obturator muscles [5]. Thus, a single incision sling (SIS) procedure was introduced to reduce the morbidity associated with anti-continence surgery and to provide outpatient office-based options to treat stress urinary incontinence [6,7,8,9]. The blind nature of needle passage, during trans-obturator tape (TOT) and tension-free vaginal tape procedures, through the retropubic (TVT) space or the obturator foramen, respectively, is associated with a relatively small risk of bladder, vascular, or nerve injury [5]. Using shorter single-incision slings is believed to reduce this risk while at the same time maintaining the same success rates of the TVT or TOT, and therefore, SIS should improve efficacy and avoid complications, such as less postoperative pain and blood loss [6,7,8,9].

A meta-analysis study comparing SISs and mid-urethral slings (MUSs) from 26 randomized controlled trials (RCTs) found no significant difference in the subjective and objective cure rates in an 18-month follow up period [7]. However, caution was addressed due to the heterogeneity of the trials studied [7]. In addition, although recent studies showed satisfaction rates with SIS, it was comparable to the conventional slings [8,9]. Furthermore, in a recent update to the surgical treatment of SUI using systemic reviews and a meta-analysis of RCTs, Kobashi et al. compared SIS to standard MUS and trans-obturator MUS [10]. Compared to the standard MUS, the subjective cure rates of SIS, but not the objective rates, were comparable in a 60-month follow-up period. However, women treated with SIS had similar objective and subjective cure rates compared with MUS for up to 12 months [11] and had similar subjective rates for up to 36 months [12,13]. On the other hand, in a post-marketing surveillance study comparing SIS to trans-obturator MUS over a 36-month period, treatment success, mesh-related complications, and serious adverse events, including pain during intercourse, pelvic pain, and urinary retention, were similar between treated groups [14].

Several SIS procedures are currently used to treat SUI, such as Altis^®^ (Coloplast, Minneapolis, MN, USA), which allows a more precise tension adjustment [8.9]. Although studies have shown the success rates of SIS in women with SUI and MUI, we are not aware of any published research using SIS to treat recurrent SUI or SUI with concomitant prolapse. Therefore, our study aimed to evaluate SIS Altis^®^ with regard to the subjective and objective cure rates of SUI, MUI, recurrent SUI, and SUI with concomitant prolapse procedures and report its complications from one single medical hospital with an average follow-up of 19 months.

## 2. Patients and Methods

### 2.1. Study Design

A retrospective cohort, unsponsored, study was conducted at the Jordan University Hospital following the protocol approval from the Institutional Review Board at the Jordan University Hospital (10/2020/8572). A single senior urogynecologist (AQ) performed all the procedures and who had previous experience placing the TVT-Secur^®^ mini-sling and MiniArc^®^.

### 2.2. Patients

One hundred and eleven women with primary SUI, recurrent SUI, or MUI treated with SIS Altis^®^ (Coloplast Corp., Minneapolis, MN, USA) between 1 June 2016 and 1 June 2019, were included. The inclusion criteria were clinically confirmed SUI, MUI, patients who had failed to respond to at least three months conservative therapies (fluid management, bladder training and antimuscarinic treatments), and recurrent SUI. The diagnosis of each condition was performed according to the guidelines by the International Continence Society (ICS) and the International Urogynecological Association (IUGA) on SUI and pelvic floor symptoms and dysfunction [15,16,17]. Patients with recurrent SUI were previously operated on due to urinary incontinence using retropubic TVT and trans-obturator TOT. A concomitant prolapse procedure was also completed as necessary.

### 2.3. Data, Clinical Examination, and Follow-Up

The patient’s data from 1 August 2020 to 30 April 2021 were assessed. Demographic variables including age, parity, body mass index (BMI), and menopausal status were collected. For statistical purposes, we divided patients into four groups, primary SUI, MUI, recurrent SUI, and patients who underwent concomitant vaginal repair surgery.

All patients were preoperatively assessed using urogynecological history and cough stress test. The cough stress test was performed by placing 300 mL of water in the bladder and asking the patient to cough; if patient leaked during the test, it was reported as a positive test. Intraoperative and postoperative complications were recorded. Complications were pain at the site of the implant, infection, mesh extraction, and sling revision. Concomitant procedures were performed and included vaginal hysterectomy, the repair of cystoceles, and rectoceles.

Patients were evaluated at 2 and 6 weeks, and then every six months as follow-up. The subjective cure is defined as the patient reporting no urine leakage when coughing, sneezing, or laughing at every visit. The objective cure is defined as the absence of urine leakage during the cough stress test performed on every follow-up visit. If during the last follow-up visit, a patient reported urine leakage or a positive cough stress test was recorded, the subjective or objective cure was considered to have failed.

### 2.4. Surgical Technique

Altis^®^ (Coloplast) sling is made of a macroporous, knitted monofilament polypropylene mesh of 7.7 cm in length. It has low elasticity (7.5%) that maintains integrity under pressure. It has two anchors, one static and one mobile, to allow tension adjustment during the procedure, which was performed under spinal or general anesthesia. The small sub-urethral incision was similar to the one made for tension-free vaginal tape slings. Peri-urethral sharp dissection up to the inferior pubic ramus using Metzenbaum scissors was carried out. The Altis^®^ needle and anchors advanced behind the inferior pubic ramus towards that obturator space bilaterally. The anchors were fixed at 2 and 10 o’clock positions. An intraoperative cough stress test or suprapubic pressure was performed, during which the tension was adjusted by pulling or releasing the suture to achieve desired continence. Cystoscopy was not routinely carried out.

### 2.5. Statistical Analysis

Statistical analysis was performed using the Statistical Package for the Social Sciences (SPSS), version 15 (SPSS Inc., Chicago, IL, USA). Descriptive statistics were used for the patient demographics. To evaluate SIS efficacy, the frequency of subjective and objective cure rates for women who had SUI, MUI, recurrent SUI, and SUI with concomitant prolapse were compared using chi-square analysis. A *p*-value less than 0.05 was considered statistically significant for all comparisons.

## 3. Results

### 3.1. Patient’s Characteristics

From June 2016 to June 2019, 111 women patients with a mean age of 48 ± 11.3 years old, BMI 29 ± 3.2 kg/m^2^, and mean parity of 6 ± 3 underwent a SIS operation using Altis^®^ (Table 1). Seventy-seven (70%) of the women were menopausal.

Seventy-eight patients (70%) suffered from SUI, and 33 (30%) suffered from MUI. Eighteen patients (16%), including both SUI and MUI subgroups, were previously operated on due to urinary incontinence (10 retropubic TVT and 8 trans-obturator TOT procedures). Forty-four patients (40%) had concomitant vaginal repair surgery due to cystocele repair, rectocele repair, and vaginal hysterectomy. The length of follow-up was 19 ± 9.5 months.

### 3.2. SIS Operation Outcome

**Figure 1:**
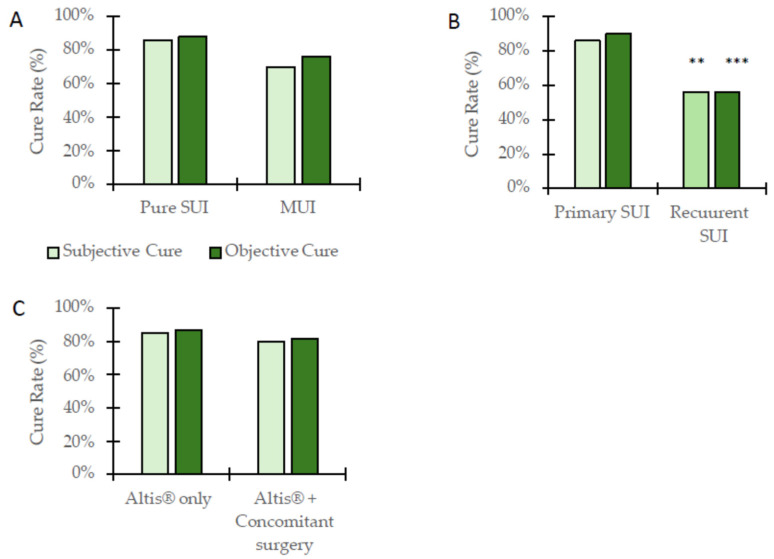
The subjective and objective cure rates of SIS (Altis^®^) performed on women with (**A**) pure SUI or MUI, (**B**) primary SUI or recurrent SUI, and (**C**) Altis^®^ only or Altis®+concomitant surgery. ** *p* = 0.003 and *** *p* = 0.0002.

The overall outcome of the SIS operation with Altis^®^ resulted in 90 patients (81%) being subjectively cured, including 67 (86%) of the SUI patients and 23 (70%) of the MUI patients (*p* = 0.046) (Table 2, Figure 1). As for the objectively cured rates, the SIS operation cured 94 patients (85%), including 69 (88%) of the SUI patients and 25 (76%) of the MUI patients. Of the 18 previously operated patients with recurrent SUI, the SIS operation resulted in 10 patients (56%) being subjectively and objectively cured (Table 2, Figure 1). These frequencies were significantly (*p* = 0.003 and 0.0002) less than the cure rates of patients that had a primary SIS operation (86% and 90%). Of the 44 patients who underwent SIS with Altis^®^ and concomitant vaginal repair surgery, 35 patients (80%) and 36 patients (82%) were subjectively and objectively cured, respectively (Table 2). These cure rate frequencies were not different than those who had the SIS procedure alone.

### 3.3. Complications of SIS

One patient had excessive bleeding of around 500 milliliters during the Altis^®^ procedure. Although the source of the bleeding could not be identified, vaginal packing for 24 h stopped the bleeding (Table 3). Three patients (2.7%) had vaginal tape erosion detected during the follow-up period, for which the surgical excision of the eroded mesh was performed. Mild pain at the site of the Altis^®^ insertion and at the groin area was reported by three patients (2.7%) during the first six weeks of the follow-up period. However, the pain was managed by simple analgesia, and disappeared entirely at the next follow-up visit. Temporary urinary retention occurred in two patients, requiring indwelling placement for one week; a urine voiding trial was repeated, and both had less than 100 mL post-void residual urine. Ten patients underwent reoperation, three for mesh erosions, two for recurrent prolapse, and five patients had retro-pubic tapes (TVT) for recurrent SUI administered (Table 3).

## 4. Discussion

In the present study, the Altis^®^ procedure for treating SUI was effective and safe during the 19 (6 to 36) months of follow-up, with overall subjective and objective cure rates of 81% and 85%, respectively. These subjective and objective cure rates may be slightly less than previous reports [9,18] because, in the present study, there were 18 women (16%) who had recurrent SUI. To our knowledge, there is no published data regarding the use of the Altis^®^ procedure in the treatment of recurrent SUI and in patients who underwent concomitant vaginal repair surgery. In the present study, patients with recurrent SUI and who underwent the Altis^®^ procedure had significantly lower subjective and objective cure rates (56% for both) when compared to patients with primary SUI (86% and 90%). Compared to other procedures, repeating MUS in recurrent SUI resulted in a 62% subjective cure rate and repeating the trans-obturator approach resulted in a 48% cure rate [19,20,21].

Our study showed that the subjective and objective cure rates using the Altis^®^ for patients with pure SUI were slightly higher than for patients with MUI. However, others have shown no differences in satisfaction between patients with MUI or SUI over 24 months after the Altis^®^ procedure [22]. Compared to MUI using traditional slings, a multicenter study showed no difference in the efficacy and safety between Atlis^®^ SIS and standard studies using trans-obturator and retropubic mid-urethral slings treating SUI and MUI [23,24]. In a multicenter study, and in the first 12-month period, only 1.1% of women experienced a device revision following the Atlis^®^ SIS procedure compared to 4.1% in the women treated with retropubic or trans-obturator MUS [24].

A randomized trial on trans-vaginal prolapse repair with or without the addition of a mid-urethral sling in women with genital prolapse and SUI confirmed that women with prolapse and coexisting SUI are less likely to have SUI after trans-vaginal prolapse repair with mid-urethral sling compared with prolapse repair only [25]. Our results showed similar continence rates of the Altis^®^ procedure if performed alone or in combination with prolapse surgery.

Only less than 10% of our patients reported a complication following the Altis procedure. Three patients (2.7%) had vaginal erosion managed definitely by mesh excision, which did not affect the continence result, and three patients (2.7%) had mild groin pain related to the Altis^®^ implant. These percentages were comparable to the mesh erosions and pain reported in other studies [9,18,21,22,23,24,25]. However, when compared with trans-obturator slings, a meta-analysis study showed that SIS had a lower postoperative score and less postoperative groin pain [26]. A recent study evaluating a 54-month follow-up period after the SIS procedure reported that 3.5% required re-operation, 4.3% had urine retention, and 3.5% had pain [27]. Except for the re-operation, these percentages are similar to those in the present study. However, in that study, the authors did not mention the cases of recurrent SUI compared to 16% in the present study.

The association of predicting factors with recurrent SUI could not be identified in the present study. One of the reasons for that is the limited cases of recurrent SUI included in the study. A recurrent SUI etiology might include factors beyond simple anatomical defects, such as neurologic conditions or mixed incontinence [28]. This complexity can limit the effectiveness of procedures like the Altis^®^ sling, designed primarily for uncomplicated stress incontinence [29]. Recurrent SUl often stems from previous surgeries or complications that alter pelvic anatomy. Scar tissue and other changes can complicate the surgical approach and reduce the procedure’s effectiveness. Studies have shown that previous surgical interventions can negatively impact outcomes in subsequent surgical treatments for incontinence [30].

The present study’s limitations are that it is retrospective, with a moderate sample size and a midterm follow period. Secondly, no standardized quality of life assessment was performed. Further research is needed with a larger sample size with a prospective design for the effectiveness of single SIS in patients with MUI and recurrent SUI.

In conclusion, the Altis^®^ procedure is effective in women who suffer from SUI, with 85% objective and 81% subjective cure rates in the 19-month follow-up period. Comparable high cure rates were seen in patients with pure SUI and MUI as well as in patients who concomitantly underwent vaginal repair. Recurrent SUI patients had lower subjective and objective success rates with the Altis^®^ procedure when compared to primary SUI patients. To confirm these results, however, further studies with larger sample sizes and prospective designs to evaluate the effectiveness of single SIS in patients with recurrent SUI are warranted.

## Figures and Tables

**Table 1:** Patient characteristics.

Parameter	Mean ± SD or N (%)
Age (years)	48 ± 11.3
Parity	6 ± 3
BMI (kg/M^2^)	29 ± 3.2
Menopause	77 (70%)
Anesthesia	
- General	40 (36%)
- Spinal	71 (64%)
SUI	78 (70%)
MUI	33 (30%)
Recurrent SUI	18 (16%)
Concomitant surgery	44 (40%)
- Cystocele repair	44 (40%)
- Rectocele repair	39 (35%)
- Vaginal hysterectomy	13 (12%)
Follow-up (months)	19 ± 9.5
- 6 to <12 months	24 (22%)
- 12 to <18 months	27 (24%)
- 18 to <24 months	24 (22%)
- 24 to <30 months	17 (15%)
- 30 to <36 months	19 (17%)

Data are given as the means ± the standard deviation or *n* (%); BMI: body mass index; SUI: stress urinary incontinence; MUI: mixed urinary incontinence.

**Table 2:** Subjective and objective cure rates of different subgroups.

Cure	Pure SUI (N 78)	MUI (N 33)	*p*
Subjective cure	67 (86%)	23 (70%)	0.046
Objective cure	69 (88%)	25 (76%)	0.089
	Primary SUI (N 93)	Recurrent SUI (N 18)	*p*
Subjective cure	80 (86%)	10 (56%)	0.003
Objective cure	84 (90%)	10 (56%)	0.0002
	Altis^®^ only (N 67)	Altis^®^ + Concomitant surgery (N 44)	*p*
Subjective cure	57 (85%)	35 (80%)	0.74
Objective cure	58 (87%)	36 (82%)	0.50

*p* value was determined using chi-square analysis.

**Table 3:** Early and late postoperative complications.

Complications	No of Patients
Excessive bleeding	1 (0.9%)
Vaginal tape erosion	3 (2.7%)
Groin pain (mild, discomfort only)	3 (2.7%)
Urinary retention	2 (1.8%)
Re-operation	10 (9%)
- Mesh erosion	3 (2.7%)
- Prolapse	2 (1.8%)
- SUI	5 (4.5%)

## Data Availability

The data presented in this study are available on request from the corresponding author upon reasonable request.
